# Soziologie im Anthropozän

**DOI:** 10.1007/s11577-022-00857-0

**Published:** 2022-10-07

**Authors:** Sebastian Schnettler

**Affiliations:** Institut für Sozialwissenschaften, Universität Oldenburg, Ammerländer Heerstraße 114–118, 26129 Oldenburg, Deutschland

## *Pries, Ludger*:

Verstehende Kooperation. Herausforderungen für Soziologie und Evolutionsforschung im Anthropozän. Frankfurt/New York: Campus Verlag 2021. 446 Seiten. ISBN: 978-3-593-51464‑2. Preis: € 34,95.

Der vorliegende Band leistet einen Beitrag zum disziplinübergreifenden Diskurs über das „Anthropozän“ (z. B. Antweiler 2022; Bajohr 2020; Block 2021; Laux und Henkel 2018). Der Begriff soll ein neues geologisches Zeitalter bezeichnen, das geprägt ist durch massive, irreversible Einwirkungen des Menschen auf die Geosphäre (Antweiler 2022). Es geht dabei nicht nur um den menschlichen Einfluss auf den Planeten, sondern um nicht weniger als die Zukunft der Menschheit selbst (z. B. Bajohr 2020; Lesch und Kamphausen 2016). Denn der Anthropos habe Probleme planetaren Ausmaßes geschaffen, ohne deren Folgen bisher komplett zu beherrschen. Diesen pessimistischen stehen optimistische Einschätzungen gegenüber, die das enorme technologische Potenzial für die Menschheit betonen, um die großen Probleme des 21. Jahrhunderts in den Griff zu bekommen (Crutzen 2006).

Pries greift diese Frage nach der Zukunft des Menschen auf und nennt mit Klimawandel, Digitalisierung, Genschere und COVID-19-Pandemie einige Beispiele für Herausforderungen im Anthropozän. Im Spannungsfeld der oben skizzierten und mehr oder weniger pessimistischen Szenarien zur Zukunft der Menschheit positioniert sich Pries, indem er vorschlägt, dass weniger technologische Innovationen als vielmehr sozialkulturelle Innovationen darüber entscheiden dürften, wie gut die Menschheit die zahlreichen Herausforderungen des 21. Jahrhunderts meistern wird. Denn, so eine zentrale These des Buches, „die bewusste soziale Gestaltung der Kultur, also der Formen menschlichen Zusammenlebens, [habe] nicht ähnlich revolutionäre Fortschritte gemacht wie die technische Naturgestaltung.“ Mögliche Entwicklungspfade im Anthropozän hängen aber auch davon ab, so eine weitere zentrale These des Buches, ob sich in der aktuellen „multipolaren Welt mit verschiedenen Spielarten von Kapitalismus, liberal-demokratischen und autoritär-populistischen Staaten“ eher wieder nationalstaatlicher Wettbewerb und sozialdarwinistisches Denken breitmache, oder ein ganzheitlich, planetarisches und auf trans- und supranationaler Kooperation beruhendes Denken durchsetze.

Um mögliche Entwicklungspfade der Zukunft zu eruieren, gehe es zunächst darum, den Menschen zu verstehen. Dies, so Pries, gelinge nur, wenn sich die Soziologie stärker gegenüber biologisch-evolutionären (bio-evo) Forschungsperspektiven auf den Menschen öffne. Denn aus seiner Sicht sind die sozialkulturellen Herausforderungen des 21. Jahrhunderts weder allein mit dem Wissensstand der Soziologie noch allein mit dem der Lebenswissenschaften zu stemmen. Es brauche, so Pries, ein evolutionssoziologisches Verständnis vom Menschen, in Abgrenzung von anderen Tierarten, um das Potenzial und die Grenzen menschlicher Kooperation zu erkunden. Ein zentrales Argument, das Pries vor dem Hintergrund der Auseinandersetzung mit der Evolutionstheorie Darwins sowie neuerer Bio-Evo-Arbeiten zum Menschen herausarbeitet, ist, dass der Mensch in komplexen, kooperativen Zusammenhängen evolviert ist. Ein Wesensmerkmal des Menschen sei sein Potenzial für verstehende Kooperation. Damit kontert Pries nicht nur sozialdarwinistische Ideen vom „survival of the fittest“, sondern schafft auch eine Brücke, die die Bio-Evo-Forschung anknüpfungsfähig für die verstehende Soziologie in der Tradition Max Webers macht.

Die zentrale Argumentationslinie des Buches wird in abgekürzter Form bereits in Kap. 1 vorgestellt. Kapitel 2 beschäftigt sich mit der allgemeinen Evolutionsforschung, inklusive Ansätzen zur kulturellen Evolution, sowie ihren Kritiken und biologistischen Verkürzungen. Pries arbeitet hier unter anderem deutlich heraus, dass eine sozialdarwinistische Denkart in Widerspruch zu modernen evolutionswissenschaftlichen Erkenntnissen über den Menschen steht. Auch wird klar, dass die moderne Forschung weit über gen-deterministische Arbeiten der frühen Soziobiologie hinausgegangen ist. In Kap. 3 zeigt Pries, dass die Soziologie wichtige Konzepte und Begriffe zu bieten hat, die die evolutionswissenschaftlichen Erkenntnisse komplementieren. Dazu führt er in kondensierter Form durch Teile der Soziologiegeschichte, um das Begriffsrepertoire der Soziologie vorzustellen. In Kap. 4 wird anhand neuerer Erkenntnisse zur Evolution des Menschen gezeigt, dass verschiedene Wissenschaftsdisziplinen im Kern zu ähnlichen Erkenntnissen zur Sozialität des Menschen gekommen sind wie die Soziologie bereits zu Beginn des 20. Jahrhunderts, wenn dort auch noch sehr allgemein formuliert. In Kap. 5 wird vor dem Hintergrund der Rezeption weiterer Bio-Evo-Forschung (z. B. zum Mensch-Tier-Vergleich und zur Ontogenese des Menschen) der Punkt stark gemacht, dass die Fähigkeiten für Sinnverstehen, Sprache und komplexe Empathie dem Menschen eigen sind. Die Evolution dieser besonderen Fähigkeiten des Menschen ließen sich dadurch erklären, dass in der menschlichen Evolution über lange Zeiträume komplexe Kooperationszusammenhänge überlebenswichtiger waren als Konkurrenzsituationen.

In Kap. 6 präsentiert Pries sein VESPER-Modell, das die in den vorigen Kapiteln vorgestellten interdisziplinären Erkenntnisse zusammenfasst und die Komplexität menschlichen Welterlebens in kondensierter und eindrücklicher Weise vor Augen führt. Pries arbeitet hier auch die zunehmende Komplexität in den letzten 150 Jahren heraus, die dem Menschen heute über den Kopf zu wachsen drohe. Institutionen ersetzen laut Pries beim Menschen Instinkte und strukturieren das menschliche Zusammenleben und Handeln. Allerdings reichen die bestehenden sozialen Institutionen nicht mehr aus, um diese Komplexität einzuhegen. Dieser Punkt wird in Kap. 7 weiter ausgeführt, auch in Konfrontation des Modells mit verschiedenen soziologischen Zeitdiagnosen. Das Buch endet mit einer deutlichen Warnung: „Ohne einschneidende und schnelle sozialkulturelle, vor allem institutionelle Innovationen wird es keine humane Zukunft geben …“.

Das Buch ist in großen Teilen eine beeindruckende Reise durch die Geschichte der Evolutionswissenschaft (sowie deren Rezeption in der Soziologie), verschiedene Teilbereiche der Lebenswissenschaften und die Geschichte der Soziologie. Mit dem Plädoyer für eine stärkere Öffnung der Soziologie für eine Bio-Evo-Perspektive auf den Menschen sowie dem überzeugenden Nachweis, dass viele der soziologischen Vorbehalte gegenüber Bio-Evo-Erklärungen auf die modernen lebenswissenschaftlichen Ansätze nicht mehr zutreffen, leistet Pries einen wichtigen Beitrag. Denn die Erkenntniszugewinne in diesen außersoziologischen Forschungsgebieten in Bezug auf soziologisch höchst relevante Themenbereichen sind groß, gleichzeitig werden diese aber kaum rezipiert, da große Teile des soziologischen Mainstream diesen Ansätzen gegenüber kritisch eingestellt bleiben (vgl. Schnettler 2016).

Überzeugend sind auch Pries’ Ausführungen zu den Herausforderungen für die Menschheit im Anthropozän sowie zur Notwendigkeit sozialkultureller und institutioneller Anpassungen. Bei all der Betonung „verstehender Kooperation“ geht jedoch ein wenig unter, dass der Mensch evolutionär mit widerstrebenden psychischen Mechanismen ausgestattet ist, die der Evolutionspsychologe Steven Pinker (2011) mal unsere „inner demons“ und „better angels“ genannt hat. Teile der Evolutionspsychologie sind zwar zurecht für ihre „prädiktive Promiskuität“ kritisiert worden (Freese 2007), jedoch findet sich in der Evolutionspsychologie ein für den vorliegenden Kontext interessanter Gedanke: Biologisch-evolutionärer und soziokultureller Wandel finden in unterschiedlichen Geschwindigkeiten statt; evolutionäre Anpassungen können somit in modernen Zeiten maladaptive Konsequenzen haben. Dies wird häufig anhand unserer Vorliebe für süße und fettige Speisen illustriert: früher ein Wegweiser zu hochkalorischem Essen und daher ein Überlebensvorteil, wird sie heute angesichts eines Überangebots an industriell hochverdichteten Lebensmitteln zu einer Gesundheitsgefahr. Könnte es angesichts der extremen Beschleunigung und Zunahme an Komplexität nicht zahlreiche Beispiele solcher Maladaptionen in Bezug auf soziale Mechanismen geben, beispielsweise auch solche, die auf der Makroebene zu Hindernissen für die eingeforderte und überlebenswichtige Ausweitung globaler Kooperation werden?

Pries’ Plädoyer für eine stärkere Öffnung der Soziologie für eine Bio-Evo-Perspektive aufgreifend möchte ich behaupten, dass deren Potenzial sogar größer ist als hier ausgearbeitet. Es gibt auch heute noch zahlreiche Beispiele für die Interaktion (!) von „nature“ und „nurture“. Neben dem genannten Zusammenhang zwischen sozialem Kontext und epigenetischen Prozessen wären auch das Zusammenspiel von sozialem Kontext, hormonellen Prozessen und menschlichem Verhalten, Empfinden und Sinnverstehen zu nennen sowie Gen-Umwelt-Interaktionen, die bedingte Plastizität des Gehirns und kontextspezifische, fakultative Mechanismen, auf die die evolutionäre Anthropologie und Psychologie verweisen. Diese und ähnliche Interaktionsprozesse zwischen biologischen und sozialen Mechanismen gilt es in empirischer Detailarbeit zu entschlüsseln. Dann könnten die Erkenntnisse daraus ggf. der fehlende Schlüssel für ein besseres und evidenzbasiertes Verständnis von Interventionsmöglichkeiten sein, die die nötigen sozialkulturellen und institutionellen Innovationen im Sinne einer humaneren Zukunft im Anthropozän anregen könnten. Das vom Autor entwickelte VESPER-Modell mag hier heuristisches Potenzial aufweisen, um solch detaillierten empirischen Arbeiten den Weg zu weisen.
